# Behavior of echocardiographic parameters of right ventricular function after tricuspid surgery

**DOI:** 10.1038/s41598-022-24048-1

**Published:** 2022-11-14

**Authors:** Diego José Rodríguez Torres, Lucía Torres Quintero, Diego Segura-Rodriguez, Jose Manuel Garrido Jimenez, Maria Esteban Molina, Francisco Gomera Martínez, Eduardo Moreno Escobar, Rocío García Orta

**Affiliations:** 1 University of Granada, Calle Fuente de Lima, N 27, 2 A, 28024 Madrid, Granada, Spain; 2 Hospital Universitario Virgen de Las Nieves, Avenida de Las Fuerzas Armadas, 2, 18014 Granada, Spain; 3 Hospital Universitario San Cecilio, Granada, Spain

**Keywords:** Cardiology, Diseases, Health care, Health occupations, Medical research

## Abstract

Evaluation of right ventricular (RV) function after tricuspid valve surgery is complex. The objective was to identify the most appropriate RV function parameters for this purpose. This prospective study included 70 patients undergoing cardiac and tricuspid valve (TV) surgery. RV size and function parameters were determined at 3 months and 1-year post-surgery. Categorical variables were analyzed with the McNemar test and numerical variables with the Student’s t-test for related samples or, when non-normally distributed, the Wilcoxon test. Spearman's rho was used to determine correlations between variables at 3 months and 1 year. RV diameters were reduced at 3 months post-surgery and were then unchanged at 1 year. Tricuspid annular plane systolic excursion (TAPSE) and S′ wave values were worse at 3 months and then improved at 1 year (t-score-2.35, *p* 0.023; t-score-2.68; *p* 0.010). There was no significant reduction in free wall longitudinal strain (LS) or shortening fraction (SF) at 3 months (t-score 1.421 and − 1.251; *p* 0.218 and 0.172), and they were only slightly below pre-surgical values at 1 year. No relationship was found between RV function parameters and mortality or major complications. During the first few months after TV surgery, LS may be a more appropriate parameter to evaluate global ventricular function in comparison to TAPSE. At 1 year, good correlations are observed between TAPSE, S′ wave, and LS values.

## Introduction

Scant data are available on the course of right ventricle (RV) size and function after tricuspid valve (TV) surgery. The RV has been the ignored ventricle for decades because it was considered to be improved by the treatment of left disease and to play a minor role in the prognosis^1–4^. However, RV dysfunction is now recognized as one of the main causes of morbidity and mortality associated with TV surgery, and its early treatment is recommended^5^.

Given the anatomical and functional complexity of the RV, no single parameter can be used for its global evaluation and multiple parameters must be considered, most frequently the tricuspid annular plane systolic excursion (TAPSE). The TAPSE is the longitudinal contraction at free margin level and represents the shift of the tricuspid annulus between end diastole and end systole. It can be readily and reproducibly measured by echocardiography but assesses only a single part of the RV and is dependent on the preload, and both shortcomings are also shared by the tissue Doppler-derived S’ wave and shortening fraction (SF)^6–11^. These limitations have been overcome in part by the development of myocardial deformation techniques that assess more segments and are less preload-dependent^12–18^.

Postsurgical RV dysfunction of unclear etiology and uncertain prognosis is a frequent finding^19^. Tamborini et al. measured the TAPSE in patients undergoing cardiac surgery and described a decrease in longitudinal contraction a few minutes after pericardiotomy^3,20^. Zanobini et al. studied patients undergoing mitral valve surgery by vertical or lateral pericardiotomy and reported that the TAPSE was altered in the former^21^. It has been postulated that the pericardium provides the RV with support for its longitudinal contraction, in which case the function would be compensated for by an increase in remaining components, keeping the global RV function within normal ranges. However, this remains controversial, and other mechanisms have been proposed to underl intrinsic myocardial dysfunction, including cardiac stunning, cardioplegia, and myocardial hypothermia, among others.

Although RV function affects the prognosis of heart surgery patients, scant data are available on the course of RV function and size during the first year after surgery^22–26^. Our hypothesis was that not all parameters show good correlations with each other during the postoperative period. The objective of this investigation was to study RV size and function at 3 months and 1 year after TV surgery and to identify the most useful echocardiographic parameters for evaluation.

## Methods

This prospective, observational single-center study included 70 patients in a third level hospital undergoing cardiac and TV surgery from February 2018 through February 2020.

Surgery was indicated in patients with heart disease (e.g., coronary or valve disease, etc.) who fulfilled the criteria for surgery in clinical practice guidelines. We confirm that all experiments were performed in accordance with relevant guidelines and regulations. At the same time, TV surgery was selected according to an established protocol based on clinical and echocardiographic variables (Supplementary material, Table 1). The TV surgery of choice was simple annuloplasty (de Vega or rigid ring), with the type being selected by the attending surgeon. Extended valve repair (e.g., anterior leaflet augmentation, neochord implantation, etc.) was performed when the tricuspid valve was distorted. If repair techniques were not feasible, a prosthesis was implanted. Patients aged > 80 years or with a life expectancy of < 2 years were excluded from the study.

The study was approved by the ethics and research committee of Virgen de las Nieves hospital. Written informed consent was obtained from all patients. Data were collected on sociodemographic, clinical, and echocardiographic variables, measuring RV size as its basal and mid diameters and end-systolic area and RV function according to TAPSE, LS, S' wave, SF, and 3D analysis results, as recommended in consensus guidelines^10^. RV size and function were evaluated before surgery and at 3 months and 1 year post- surgery. The study was carried out with a GE Vivid 9 machine and EchoPAC software, applying Speckle Tracking 2D Strain. An RV-focused apical 4-CH view was used for the analysis.

Software SPSS (version 16) was used for statistical analyses. The normality of data was checked with the Kolmogorov–Smirnov test. A descriptive analysis was performed, expressing quantitative variables as means ± standard deviation when normally distributed and medians ± range when not, and calculating number and percentage for qualitative variables. Categorical variables were analyzed with the McNemar test and numerical variables with the Student’s t-test for related samples or, when non-normally distributed, with the Wilcoxon test. Spearman's rho coefficient was used to analyze correlations between variables (TAPSE, LS, S' wave, and SF) at 3 months and 1 year.

## Results

The etiology of TI was functional in 69.9% of patients, rheumatic in 20.5%, myxomatous in 2.7%, pacemaker-mediated in 1.4%, and due to radiation in 1.4%. Previous RV function was normal in 82.2%, mildly depressed in 13.7%, and moderately depressed in 4.1% (Table 1).

**Table 1:** Baseline characteristics.

**Baseline characteristics**	
Age (years)	65.47 ± 10.3
Body mass index (kg/m^2^)	28.06 ± 4.17
Sex (female)	53/75.70
Hypertension (n/%)	38/56.20
Diabetes (n/%)	9/12.3
Glomerular filtration rate (ml/min/m^2^)	72.81/–18.45
Chronic obstructive pulmonary disease (n/%)	12/17.40
Dyslipidemia (n/%)	30/43.80
**Cardiovascular history**	
NYHA functional class > 2 (n/%)	30/42.80
Right heart failure (n/%)	32/46.40
Preoperative atrial fibrillation (n/%)	55/79.50
Coronary disease (n/%)	6/8.6
Stroke (n/%)	9/12.8
PASP (mmHG)	52.45 ± 15.96
PASP (n/%)	15/21.43%
**LVEF (n/%)**	
> 52% in males or > 54% in females	56/80.0
41%–51/53% (males/females)	10/14.2
30%–40%	3/4.2
< 30%	1/1.4
**RVEF (n/%)**	
TAPSE > 17 mm	57/81.4
15–17 mm	10/14.2
< 15 mm	3/4.2
**Mitral valve etiology (n/%)**	
	Rheumatic 28/40%
	Fibroelastic degeneration 7/10%
	Myxoid 7/10%
	Myocardiopathy 3/4.8%
	Previous prosthesis 13/18%
	Other causes 12/17.1%
**Tricuspid valve etiology (n/%)**	
	Functional 49/69.9
	Rheumatic 14/20.5
	Myxomatous 2/2.7
	Pacemaker-mediated 1/1.4
	Radiation-mediated 1/1.4
	Unspecified 3/4.1
**Type of valve surgery (n/%)**	
	Tricuspid alone 17/24.7
	Tricuspid + Mitral 30/42.5
	Tricuspid + aortic 2/2.7
	Tri-valvular 14/20.5
	Other type 7/9.6

NYHA: New York Heart Association; PASP: pulmonary artery systolic pressure; LVEF: left ventricle ejection fraction; RVEF: right ventricle ejection fraction.

Rigid ring annuloplasty was performed in 30.1% of patients, De Vega annuloplasty in 23.3%, extensive repair (e.g., anterior leaflet augmentation, neochord implantation, etc.) in 9.6%, and prosthetic replacement in 21.9%; 94.5% underwent valve surgery alone and the remainder in combination with coronary or other surgery. The type of valve surgery was mitral in 42.5% of patients, aortic in 2.7%, trivalvular (aortic, mitral, and tricuspid) in 20.5%, tricuspid alone in 24.7%, and another type of surgery in 6.8%.

Before surgery, TI was mild in 9.5% of patients, moderate in 32.1%, moderate-severe in 9.5%, and severe in 39.3%. At 1 year, it was mild in 45.2%, moderate in 4.8%, moderate-severe in 3.2%, severe in 1.6%, and trivial or absent in 45.2%. Pulmonary artery systolic pressure was 51.56 mmHG ± 13.79 mmHG before surgery and 41.82 mmHG ± 11.60 mmHG at 1 year.

Table 2 shows RV size measurements before surgery and at 3 months and 1 year post-surgery. Basal and mid diameters were reduced at 3 months and were then unchanged at 1 year (t basal diameter 9.55 *p* < 0.001 and t mid diameter 5.32 *p* < 0.001). Similar results were observed for the end-systolic/diastolic area, although the difference did not reach statistical significance.

**Table 2:** RV size measurements before surgery and at 3 months and 1 year post-surgery.

	Presurgical	Three months	Sig	One year	Sig
Basal diameter (mm)	49.13 ± − 7.83	40.84 ± 4.40	.012	39.92 ± − 5.24	.001
Mid diameter (mm)	38.06 ± − 8.66	32.76 ± 5.13	.001	32.6 ± − 5.49	.001
End-systolic area (cm^2^)	20.34 ± − 13.53	14.38 ± 4.23	.086	14 ± − 5.21	.606
End-diastolic area (cm^2^)	33.22 ± 15.28	25.87 ± 5.61	0.7	24.96	0.6
Tricuspid annulus diameter (mm)	41.77 ± − 7.41	33.78 ± 5.07	.000	35.66 ± − 6.95	.000
TAPSE (mm)	19.08/− 4.49	14.38 ± 4.23	0.02	16.7 ± − 3	0.09
Fractional change area (%)	39.84 ± -13.67	38.02 ± 12.60	0.6	39.49 ± − 11.16	0.01
Tricuspid lateral systolic peak S’ wave velocity (m/s)	0.11 ± − 0.31	0.08 ± 0.020	.001	0.09 ± − 0.029	.000
RV free wall longitudinal strain (%)	− 19.83 ± − 4.72	-17.92 ± − 4.79	.816	− 17.7% ±—5.14%	.814
RA longitudinal diameter (mm)	71.6 ± − 13.71	54.94 ± − 9.46	.000	53.34 ± − 11.59	.000
RA transversal diameter (mm)	53.11 ± − 13.17	43.55 ± − 7.44	.000	44.34 ± -10.41	.000
RA indexed biplane volume (ml/m^2^)	673.9 ± − 48.51	41.57 ± − 19.89	.001	45.78 ± − 24.59	.000
Vena contracta (mm)	8.09 ± − 3.63	2.68 ± − 1.9	.000	1.78 ± − 2.26	.000
PISA radius (mm)	7.64 ± − 3.08	0.96 ± − 2.2	.000	0.97 ± − 1.95	.000
Regurgitating orifice (cm^2^)	0.74 ± − 0.46	0.03 ± − 0.1	.000	0.09 ± − 0.24	.000
Regurgitating volume (ml)	44.82 ± − 26.98	5.02 ± − 17.1	.000	8.73 ± − 19.14	.000

RV, right ventricle; RA, right atrium; TAPSE, tricuspid annular plane systolic excursion.

**Figure 1:**
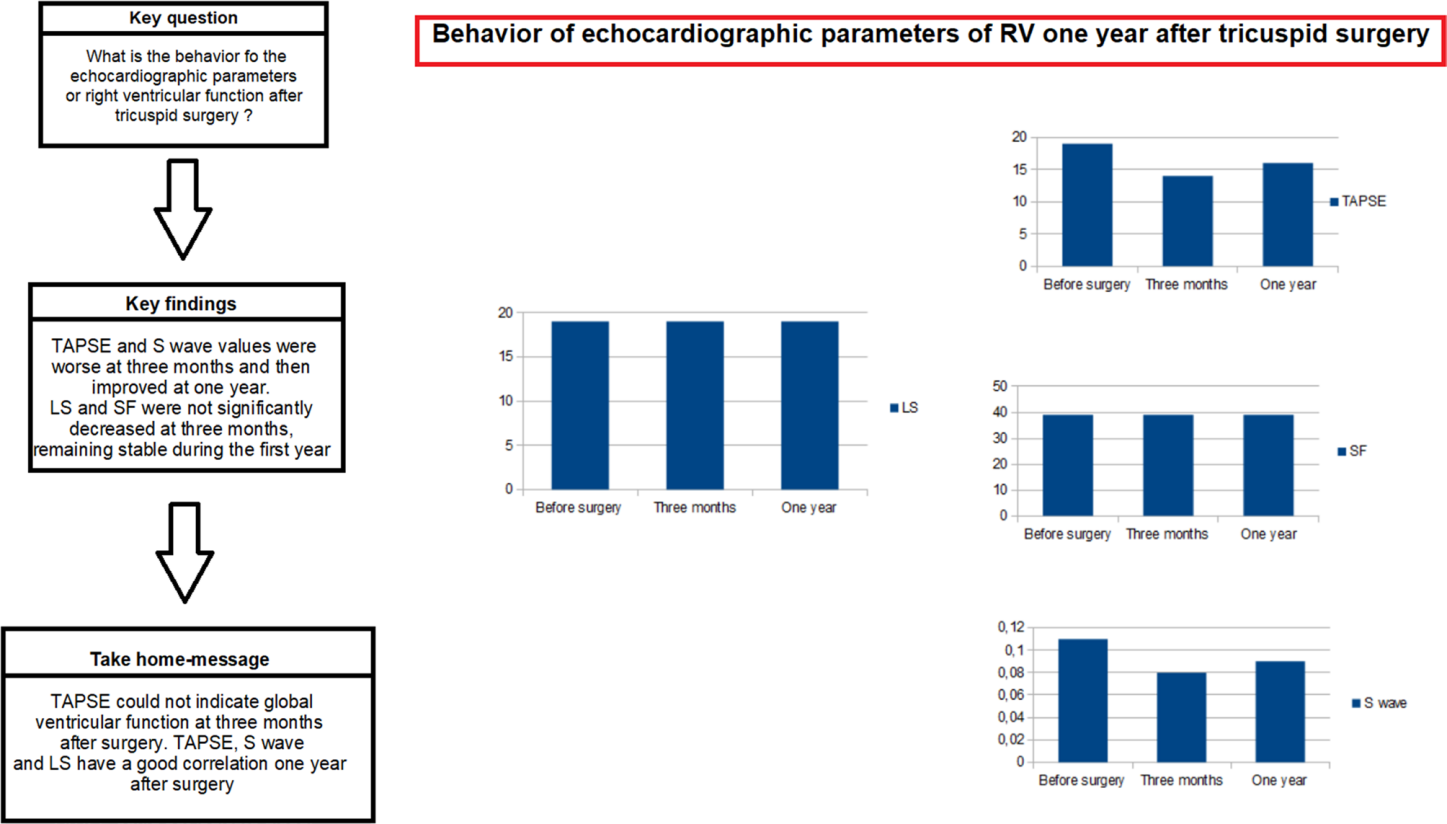
Behavior of the function of the right ventricle after tricuspid valve surgery.

TAPSE and S' wave results for RV function were worse at 3 months (t − 2.35 p 0.023) and were then improved at 1 year but without reaching presurgical values (t − 2.68; p 0.010). There was no statistically significant change in SF, while global GLS evidenced a non-significant trend towards a slight decrease at 3 months and then remained stable at 1 year (t 1.42 p 0.17) (Fig. 1).

Sub-analyses revealed that the presurgical RV size was progressively larger with greater volume overload from mild to moderate-severe and severe TI, and that the SF increased from mild to moderate-severe TI but was then significantly decreased in severe TI (Fig. 1). No significant differences were found in the remaining parameters, although tendencies were observed towards a progressive decrease in TAPSE from mild to moderate-severe TI followed by an increase with severe TI, towards a decrease in S’ wave from mild to severe TI, and towards a progressive increase in GLS from mild to moderate-severe TI followed by a decrease with severe TI (Table 3).

**Table 3:** TAPSE, Sl, SF, and S’ wave according to TI grade.

	SF (%)	TAPSE (mm)	S' wave (m/s)	Longitudinal strain (%)
Mild	27.71	19.57	0.12	− 19.57
Moderate	40.41	19.39	0.12	− 20.00
Moderate-severe	45.91	17.75	0.10	− 22.75
Severe	40.79	19.14	0.10	− 19.29

SF, shortening fraction; TAPSE, tricuspid annular plane systolic excursion.

The RV ejection fraction (RVEF) was compared by visual estimation before surgery and at 1 year, observing significant differences (Z-2.71 p 0.007). An improvement was observed in four patients, from mild-moderately depressed to normal RVEF, and a worsening in 16 patients, from normal to mildly depressed (n = 13), normal to severely depressed (n = 1), and mild-moderately to severely depressed RVEF (n = 2). No changes were observed in the remaining patients, most of whom had a normal RVEF before surgery and at 1 year.

Spearman’s rho results evidenced a good correlation between TAPSE and S' wave at 3 months (r 0.62, p 0.001), when LS was poorly correlated with S' wave and SF values (r – 0.36 p 0.11; r − 0.47 p 0.17). No correlation was found between TAPSE and LS (r − 0.36; p 0.051). At 1 year, a good correlation was found between all parameters.

No differences in RV size or function were observed as a function of the repair technique (rigid ring or De Vega annuloplasty or extensive repair). RV size was larger and its function worse at 1 year after prosthetic replacement than after repair by any technique; however, these differences were already present before the surgery (Table 2 Supplementary material).

## Discussion

Assessment of RV function continues to be challenging because of the geometry of the RV and the little information available in specific settings (e.g., post-surgery). It also remains controversial whether the longitudinal function of the RV represents its global systolic function. Further, the presence of overloaded and hyperfunctioning ventricles before surgery may lead to an overestimation of RV function. Tamborini et al. reported that a postoperative decrease in the TAPSE was not a good indicator of global function, which was found to be normal in 3D echocardiograms^10^. In the present study, TAPSE and S' wave values were significantly decreased at 3 months and were then recovered at 1 year. This initial behavior was not observed for LS or SF values, although the values of all studied parameters almost reached normality at 1 year. TAPSE and S’ wave values might not be good indicators of RV function at 3 months post-surgery, when they showed no correlation with LS. The early decrease in TAPSE and S’ wave values might not represent a real decrease in RV function but rather an alteration in longitudinal displacement compensated by an increase in circumferential contractility, consistent with the lack of significant changes in strain.

LS showed a slight decrease at 3 months, but the difference was not statistically significant, suggesting that this parameter has a lesser variability. This could indicate that the technique of choice during the early postoperative period is LS measurement. All three parameters proved valid to evaluate function at 1 year, when they all indicated that it was at the lower limit of normality or mildly depressed.

Strain estimation is highly dependent on the echocardiography equipment and methodology used, and variations in these can result in significant changes. The same equipment was always used in the present study, following recommended reference guidelines^8,10–13,15,27^. A decision was taken to consider the LS rather than the global strain due to the high proportion of patients undergoing left surgery, allowing a more specific assessment of RV function.

3D echocardiography has gained acceptance for the evaluation of RV volumes and EF. Several studies have shown its feasibility, accuracy, and reproducibility, mainly in single-center studies or in centers with high level of expertise. However, the 2015 Guidelines on cardiac chamber quantification recommend this approach in cases of good image quality and expertise as the preferred modality to assess RV function^6^. Moreover, even when the data show the normal reference values for RV morphology and function, the prognostic impact of 3D RVEF in different cardiac conditions is unclear. Image quality is usually limited in postoperative patients, especially in those with implanted prosthetic valves and/or atrial fibrillation. In the present study, all patients underwent tricuspid valve surgery followed by three-dimensional systematic acquisition of the right ventricle. However, the image quality was considered insufficient for accurate assessment by this method in a substantial number of patients.

Right ventricular function was assessed using a multiparameter approach, both qualitative and semiquantitative, given the absence of a single parameter that accurately reflects right ventricular function, especially in postoperative patients. One possible assessment of right ventricular function is through visual estimation by an expert echocardiographer, but the lack of precision only allows differentiation between normal function and mild or advanced dysfunction. Several cut-off points were selected for different parameters to define right ventricular dysfunction, which was considered if at least two of the following parameters were present (TAPSE: mildly depressed: 15–17 mm, moderately to severely depressed: < 15 mm; SF: depressed < 35%; DTI: depressed < 9.5 cm/s; Global longitudinal RV free wall strain, depressed < 20%) and coincided with the visual estimation.

One limitation of the conventional parameters is their dependence on afterload. Different afterloads can explain distinct longitudinal strain values ​in patients with similar ventricular function. Right ventricular myocardial work (RVMW) takes account of afterload and yields information on the coupling between the RV and pulmonary artery, which could provide a more accurate estimate of RV systolic function. RVMW is a novel method for the non-invasive assessment of RV function using RV stress-pressure loops. It may offer new insights into the role of the RV in pulmonary hypertension. It provides a comprehensive analysis of RV systolic function and correlates more closely with invasively measured stroke volume and stroke volume index in comparison to other standard echocardiographic parameters. Unlike RV longitudinal strain, TAPSE, and SF, RVMW parameters integrate contractility, RV desynchrony, and pulmonary pressures in the quantification. In addition, RVMW is not subject to the technical limitations of other standard RV systolic function parameters^28–31^. Patients were recruited from February 2018 to February 2020 in the present study, and there was no estimation of myocardial work.

In the present study, a proportionally greater RV dilatation was observed with more severe TR, finding an increase in RV basal and mid diameters with a higher degree of TR (p < 0.001). After correcting for the TR, RV size was found to be decreased at 3 months post-surgery and then remain stable at 1 year. In relation to function parameters, the SF significantly and progressively increased with higher TR grade from mild to moderate-severe and then decreased with severe TR; however, this is not likely to represent the global function, given the high dependence on volume. No statistically significant differences with presurgical values were found for the other function parameters, observing only non-significant tendencies. These findings may suggest that neither that TAPSE nor SF are valid for assessment in patients with severe TR because of their volume dependence, although TAPSE may be relatively independent from the degree of TR and more related to RV function, except in cases of severe TR. S' wave showed minimal variations and may be less dependent on volume. LS progressively increased with higher TR grade and then decreased with severe TR, reflecting a worse RV function with more severe TR and a lesser dependence on volume Very little evidence has been published on the validity and limitations of RV function and size parameters for assessment in the early postoperative period. Studies with larger samples and longer follow-up periods are required to elucidate the role and limitations of imaging techniques for this purpose.

## Limitations

RV volumes were acquired in three dimensions for ventricular function analysis, but the quality of images was not considered adequate for a precise assessment. The echocardiographic window for RV estimation is limited in patients with previous surgery, and many of the present patients had undergone multiple surgeries.

No other reference technique (e.g., magnetic resonance imaging) was applied to study ventricular function, because the main objective was to follow the time course of echocardiographic parameters for 1 year after the surgery. In addition, MRI is a low-availability technique that is not always recommended in recently operated patients. The high proportion of patients with prostheses in mitral and aortic position and pacemakers would add complexity to the analysis. However, the application of magnetic resonance imaging, for example, would have allowed the comparison of function between baseline and 1-year post-surgery to verify the hypotheses based on echocardiographic parameters.

RV function parameters were slightly decreased at 1 year but did not reach presurgical values, and it is possible that they might have normalized over the longer term. Studies with longer follow-up periods are required to explore this possibility.

## Conclusions

During the first few months post-surgery, LS may be a valid parameter for RV function assessment but TAPSE and S’ may not be, due to possible changes in longitudinal function. At 1-year post-surgery, all parameters appear valid to estimate global RV function. For pre-surgical assessment, TAPSE and SF appear to be the most dependent on volume and may not be useful in patients with severe TR, for whom LS, which is less volume-dependent, may be a valid parameter.

## Supplementary Information

Supplementary Information.

## Data Availability

All data generated or analyzed during this study are included in this published article [and its supplementary information files].

## Abbreviations

RV: Right ventricle
TI: Tricuspid insufficiency
TAPSE: Tricuspid annular plane systolic excursion
LS: Free wall longitudinal strain
S: S′ wave peak by tissue Doppler
SF: Shortening fraction
LV: Left ventricle
RVEF: Right ventricle ejection fraction

## References

[1] Singh JP, Evans JC, Levy D (1999). Prevalence and clinical determinants of mitral, tricuspid, and aortic regurgitation. Am. J. Cardiol..

[2] Sanz J, Sánchez-Quintana D, Bosson D, Bogaar H, Naeije R (2019). Anatomy, function, and dysfunction of the right ventricle: JACC state-of-the-art review. J. Am. Coll. Cardiol..

[3] Haddad F, Hunt SA, Rosenthal DAN, Murphy DJ (2008). Right ventricular function in cardiovascular disease, part I: Anatomy, physiology, aging, and functional assessment of the right ventricle. Circulation.

[4] Ho SY, Nihoyannopoulos P (2006). Anatomy, echocardiography, and normal right ventricular dimensions. Heart.

[5] Badano LP, Addetia K, Pontone G (2020). Advanced imaging of right ventricular anatomy and function. Heart.

[6] Rudski LG, Lai WW, Afilalo J (2010). Guidelines for the echocardiographic assessment of the right heart in adults: A report from the American Society of Echocardiography Endorsed by the European Association of Echocardiography, a registered branch of the European Society of Cardiology, and the Canadian Society of Echocardiography. J. Am. Soc. Echocardiogr..

[7] Lang RM, Badano L, Mor-Avi V (2015). Recommendations for cardiac chamber quantification by echocardiography in adults: An update from the American Society Echocardiography and the European Association of Cardiovascular Imaging. J. Am. Soc. Echocardiogr..

[8] Badano LP, Muraru D, Parati G, Haugaa K, Voigt JU (2020). How to do right ventricular strain. Eur. Heart J. Cardiovasc. Imaging.

[9] Galderisi M, Cosyns B, Edvardsen T (2017). Standardization of adult transthoracic echocardiography reporting in agreement with recent chamber quantification, diastolic function, and heart valve disease recommendations: An expert consensus document of the European Association of Cardiovascular Imaging. Eur. Heart J. Cardiovasc. Imaging.

[10] Voigt JU, Pedrizzetti G, Lysyansky P (2015). Definitions for a common standard for 2D speckle tracking echocardiography: consensus document of the EACVI/ASE/industry task force to standardize deformation imaging. J. Am. Soc. Echocardiogr..

[11] Andersen MV, Moore C, Sogaard P (2019). Quantitative parameters of high-frame-rate strain in patients with echocardiographically normal function. Ultrasound Med. Biol..

[12] Joos P, Poree J, Liebgott H (2018). High-frame-rate speckle-tracking echocardiography. EEE Trans. Ultrason. Ferroelectr. Freq. Control..

[13] Furlani AC, Garcia MJ (2019). Right ventricular strain. Circ. Cardiovasc. Imaging.

[14] Smolarek D, Gruchala M, Sobiczewski W (2017). Echocardiographic evaluation of right ventricular systolic function: The traditional and innovative approach. Cardiol. J..

[15] Samarai D, Ingemansson SL, Gustafsson R, Thilén U, Hlebowicz J (2020). Global longitudinal strain correlates to systemic right ventricular function. Cardiovasc. Ultrasound.

[16] Lakatos B, Kovács A, Tokodi M, Doronina A, Merkely B (2016). Assessment of the right ventricular anatomy and function by advanced echocardiography: Pathological and physiological insights. Orv. Hetil..

[17] Schneider M, Aschauer S, Mascherbauer J (2019). Echocardiographic assessment of right ventricular function: Current clinical practice. Int. J. Cardiovasc. Imaging.

[18] Addetia K, Patel AR (2014). Beyond right ventricular size and function: The importance of evaluating the right ventricle's capacity for recovery. Expert Rev. Cardiovasc. Ther..

[19] Denault A, Haddad F, Lamarche Y, Bouabdallaoui N, Deschamps A, Desjardins G (2020). Postoperative right ventricular dysfunction—Integrating right heart profiles beyond long-axis function. J. Thorac. Cardiovasc. Surg..

[20] Tamborini G, Muratori M, Brusoni D (2009). Is right ventricular systolic function reduced after cardiac surgery? A two- and three-dimensional echocardiographic study. Eur. J. Echocardiogr..

[21] Zanobini M, Saccocci M, Tamborini G (2017). Postoperative echocardiographic reduction of right ventricular function: Is pericardial opening modality the main culprit?. Biomed. Res. Int..

[22] Dutta T, Aronow WS (2017). Echocardiographic evaluation of the right ventricle: Clinical implications. Clin. Cardiol..

[23] Mandoli GE, Cameli M, Novo G (2019). Right ventricular function after cardiac surgery: The diagnostic and prognostic role of echocardiography. Heart Fail. Rev..

[24] Nguyen T, Cao L, Movahed A (2014). Altered right ventricular contractile pattern after cardiac surgery: Monitoring of septal function is essential. Echocardiography.

[25] Denault A, Haddad F, Jacobsohn E, Deschamps A (2013). Perioperative right ventricular dysfunction. Curr. Opin. Anaesthesiol..

[26] Garcia R, Carreño E, Mayordomo S (2016). Evaluation of right ventricular function after cardiac surgery: The importance of tricuspid annular plane systolic excursion and right ventricular ejection fraction. J. Thorac. Cardiovasc. Surg..

[27] Voigt JU, Cvijic M (2019). 2- and 3-dimensional myocardial strain in cardiac health and disease. JACC Cardiovasc. Imaging.

[28] Trifunovic-Zamaklar D, Vratonjic J (2022). Is noninvasive right ventricular myocardial work analysis the right way for functional assessment of the right ventricle?. J. Clin. Ultrasound.

[29] Butcher SC, Feloukidis C, Kamperidis V (2022). Right ventricular myocardial work characterization in patients with pulmonary hypertension and relation to invasive hemodynamic parameters and outcomes. Am. J. Cardiol..

[30] Butcher SC, Fortuni F, Montero-Cabezas JM (2021). Right ventricular myocardial work: Proof-of-concept for non-invasive assessment of right ventricular function. Eur. Heart J. Cardiovasc. Imaging.

[31] Wu J, Huang X, Huang K (2022). Correlations among non invasive right ventricular myocardial work indices and the main parameters of systolic and diastolic functions. J. Clin. Ultrasound.

