# Supplementary material for: Behavior of echocardiographic parameters of right ventricular function after tricuspid surgery
Source: Sci Rep. 2022 Nov 14;12:19447. doi: 10.1038/s41598-022-24048-1 (PMC9663697; doi:10.1038/s41598-022-24048-1)
Supplement: Supplementary file 1 — Supplementary Information. [file 41598_2022_24048_MOESM1_ESM.docx]

**Supplementary material**

Table 1: Tricuspid valve surgery protocol

| **VALVE REPAIR** | |
| --- | --- |
| TI grade III or IV | Always |
| TI grade I (if mitral surgery) or II/IV (if other surgery) plus one criterion out of: | Annulus > 39 mm (21 mm/m^2^)  Permanent atrial fibrillation  Mitral rheumatic valve disease  Previous right heart failure |
| **ADVANCED REPAIR (IF PROSTHESIS IS NOT POSSIBLE)** | |
| If any of the following criteria are met:  -Severe valve anatomy distortion (tenting area> 1.0 cm2 and coaptation> 8 mm)  -RV dilatation or severe dysfunction (visual EF or TAPSE < 13)  -Substantive calcification/thickening/fibrosis of leaflets or annulus | |

TI: tricuspid insufficiency; RV: right ventricle; TV: tricuspid valve; EF: ejection fraction; TAPSE: tricuspid annular plane systolic excursion.

Table 2: RV size and function results by type of tricuspid surgery

|  | Type of tricuspid surgery | Mean | P |
| --- | --- | --- | --- |
| RV basal diameter | Repair | 47.11 | 0.001 |
|  | Prosthesis | 54.15 |  |
| RV mid diameter | Repair | 36.57 | 0.026 |
|  | Prosthesis | 41.8 |  |
| RV end-systolic area | Repair | 13.95 | 0.012 |
|  | Prosthesis | 21.40 |  |
| RV end-diastolic area | Repair | 23.14 | 0.02 |
|  | Prosthesis | 35.89 |  |
| Fractional change area | Repair | 39.324 | 0.83 |
|  | Prosthesis | 40.215 |  |
| TAPSE | Repair | 19.44 | 0.58 |
|  | Prosthesis | 18.75 |  |
| S’ wave | Repair | .1209 | 0.027 |
|  | Prosthesis | .1015 |  |
| Longitudinal strain | Repair | -20.56 | 0.14 |
|  | Prosthesis | -17.91 |  |

RV: right ventricle, TAPSE: tricuspid annular plane systolic excursion
